# Evaluation Procoagulant Activity and Mechanism of Astragalin

**DOI:** 10.3390/molecules25010177

**Published:** 2020-01-01

**Authors:** Changqin Li, Miyun Hu, Shengjun Jiang, Zhenhua Liang, Jinmei Wang, Zhenhua Liu, Hui-Min David Wang, Wenyi Kang

**Affiliations:** 1 National R & D Center for Edible Fungus Processing Technology, Henan University, Kaifeng 475004, China; lcq@vip.henu.edu.cn (C.L.); humiyun@vip.henu.edu.cn (M.H.); jiangshengjun66@163.com (S.J.); liangzhenhua1998@hotmail.com (Z.L.); wangjinmeiscp@126.com (J.W.); 2 Joint International Research Laboratory of Food & Medicine Resource Function, Kaifeng 475004, China; 3 Graduate Institute of Biomedical Engineering, National Chung Hsing University, Taichung City 402, Taiwan; 4 Kaifeng Key Laboratory of Functional Components in Health Food, Kaifeng 475004, China

**Keywords:** *Rose chinensis* Jacq, Astragalin, procoagulant effect

## Abstract

Astragalin, isolated from flowers of *Rosa chinensis* Jacq., is a kind of flavonoid, with anti-inflammatory, antioxidant, antiviral, analgesic, antibacterial, antiallergic, and antihepatotoxic effects. However, no studieson the procoagulant effect of astragalin have been reported. This study aimed to investigate the procoagulant activity of astragalin and its mechanism. Its procoagulant effect was investigated by activated partial thromboplastin time (APTT), thrombin time (TT), prothrombin time (PT), and fibrinogen (FIB) in vitro, and a rat model established by heparin sodium was used to evaluate the mechanism for the procoagulant effect in vivo. The results showed that astragalin had good procoagulant effects compared with the control group in vitro. Compared with the model group in vivo, astragalin could shorten the coagulation time and significantly increase the number of platelets. Meanwhile, astragalin could significantly reduce the effectual time of PT and APTT and increase the content of FIB. The contents of 6-keto-PGF_1*α*_ and eNOS significantly decreased. Astragalin could increase whole blood viscosity (WBV), plasma viscosity (PV), erythrocyte sedimentation rate (ESR) and packedcell volume (PCV). All of the above revealed that astragalin had good procoagulant effects by promoting the intrinsic and extrinsic coagulation system.

## 1. Introduction

There are hemostatic and coagulation systems, anticoagulation, and fibrinolytic systems in human body under physiological conditions, which restrict each other and are in a dynamic equilibrium state. When abnormalities in the functioning of one system could cause bleeding or thrombosis followed by a series of diseases, such as hemophilia and vascular disease [1,2,3].

Hemophilia [4,5,6] is a group of hereditary hemorrhagic diseases with coagulation disorders. The common characteristics of hemophilia are the disorder of active thrombozyme, the prolongation of coagulation time, lifelong tendency of bleeding after minor trauma, and “spontaneous” bleeding in severe patients without obvious trauma. The treatment of hemophilia is mainly local hemostatic treatment and alternative therapy, but its cure rate is low, and there are obvious side effects, such as an increase in the risk of hepatitis, hemolysis, and acquired immunodeficiency disease (AIDS) [7,8,9].

In addition to the hemorrhagic diseases, obstetric hemorrhagic bleeding, war wound bleeding, and accidental trauma bleeding were treated with tourniquet bandage compression hemostasis. However, its side effects are obvious, such as nerve paralysis, limb injury, and local tissue necrosis when the use time is too long [10]. Moreover, hemostatic drugs can help people with war injuries or serious accident stop bleeding immediately, which is important for their subsequent therapy [11,12]. However, it is still a difficult and important task to find new hemostatic drugs. Therefore, many more studies focus on natural active ingredients due to their significant effect and lower side effects.

At present, many traditional Chinese medicines are widely used as hemostatic drugs, including *Panax notoginseng* (Burkill) F. H. Chen ex C. H., *Herba ecliptae*, *Sanguisorba officinalis* L., leaves of *Platycladus orientalis*. (L.) Franco, roots of *Rubia cordifolia* L., and the node and chamber of *Nelum bonucifera* Gaerth. Additionally, there are many compounds with hemostatic effects. Up to now, the type of active components included alkaloids, flavonoids, organic acids, coumarins, amino acids, phenols, tearers, terpenoids, and lipids. For example, isorhamnetin-3-*O*-rutin-glycoside [13] and quercetin isolated from *Sophora japonica* Linn had the effect of vasoconstriction. Agrimonine in *Agrimonia pilosa* Ldb. showed a hemostatic effect on platelets [14]. Tannins in *Daemonorops draco* Bl., catechins and gallic acid in *Rheum palmatum* L., and tannins and polyphenol in *Sanguisorba officinalis* L. could promote the activation of the coagulation system [15,16]. Pectolinarin in *Cirsium japonicum* Fisch. *E*x DC and iridoid glycoside in *Lamiophlomis rotata* (Benth.) Kudo could inhibit fibrinolytic [17,18]. Chimaphilin isolated from the whole grass of *Pyrola calliantha* H. and had procoagulant activity [19]. Isoverbascoside had a strong inhibitory effect on oxidative hemolysis in vitro [20]. Demethylwedelolactone and wedelolactone from *Hypericum* Linn showed a good hemostatic activity. It had the effect of vasoconstriction and inhibited the activity of fibrinolytic system to play a hemostatic role [21]. However, the mechanism of some active ingredients on hemostatic action is not clear.

Therefore, the flowers of *Rosa chinensis* Jacq., belonging to the Rosaceae family [22], were investigated and astragalin was identified. Astragalin (kaempferol-3-*O*-*β*-d-glucoside), is a kind of flavonoid found in many traditional Chinese herbs [23,24,25], has anti-inflammatory, antioxidant, antiviral, analgesic, antibacterial, antiallergic, and antihepatotoxic effects [26,27]. However, no studieson the procoagulant effect of astragalin have been reported. This study aimed to investigate the procoagulant activity of astragalin and its mechanism.

## 2. Results and Discussion

### 2.1. Coagulation Time Test In Vitro

#### 2.1.1. Effects on Plasma Coagulation Parameters In Vitro

**Figure 1:**
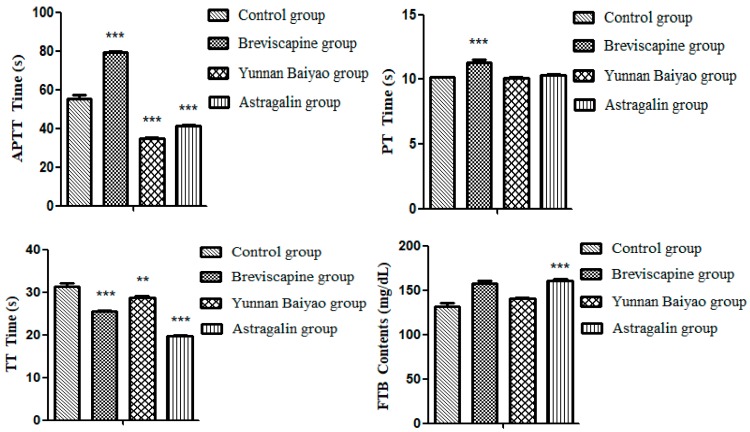
Results of the coagulation four indices of samples ($\bar{X}$ ± SD, *n* = 3). Compared with the control group: *** *p* < 0.001, ** *p* < 0.01.

Table 1 and Figure 1 showed that, the effectual time of APTT (*p* < 0.001), TT (*p* < 0.001) and FIB (*p* < 0.001) could be significantly shortened by both astragalin (2 mg/mL) and Yunnan Baiyao compared with the control group, indicating that astragalin had the same effect of coagulant activity with Yunnan Baiyao. Yunnan Baiyao and Breviscapine were positive drugs.

#### 2.1.2. Effect of Different Concentration of Astragalin on Coagulation In Vitro

**Figure 2:**
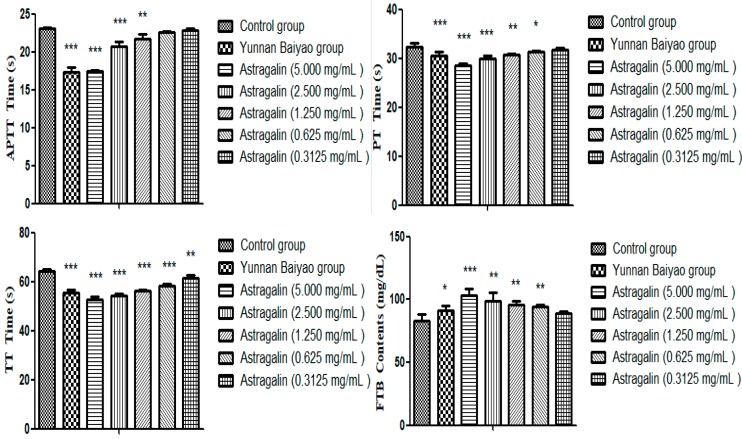
Procoagulant activity of different concentrations of astragalin ($\bar{X}$ ± SD, *n* = 3). Compared with control group: *** *p* < 0.001, ** *p* < 0.01, * *p* < 0.05.

In Table 2 and Figure 2, the astragalin group showed a dose-dependent increase compared with the control group. Except for the 0.3125 mg/mL group, APTT, PT, TT (*p* < 0.001), and FIB (*p* < 0.001) could be significantly shortened by both astragalin indicating that astragalin had a good coagulant effect, and the effect of the 5.000 mg/mL group was better than Yunnan Baiyao as a positive drug.

### 2.2. Effects on Plasma Coagulation Parameters In Vivo

#### 2.2.1. Effects of Astragalin on Coagulation Time (CT) in Rats

**Figure 3:**
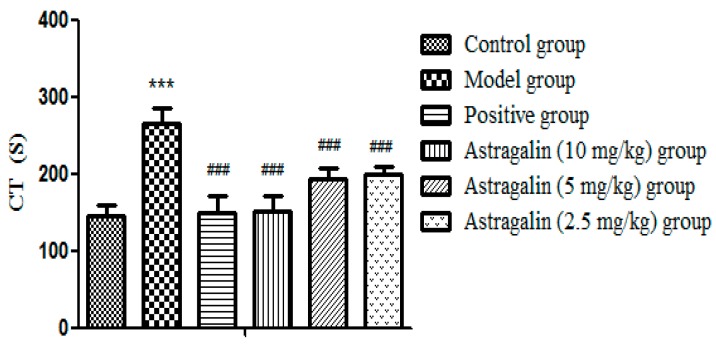
Effects of astragalin on CT (X ± SD, *n* = 8). Compared with the control group: *** *p* < 0.001; compared with the model group: ^###^ *p* < 0.001.

In Table 3 and Figure 3, CT was significantly increased in the model group after seven days of administration compared with the control group (*p* < 0.001), which suggested that model was successfully established. Compared with the model group, both the positive group and the astragalin (10, 5 and 2.5 mg/kg) group had significantly shorter CT (*p* < 0.001).

#### 2.2.2. Effects of Astragalin on Platelets (PLC)

**Figure 4:**
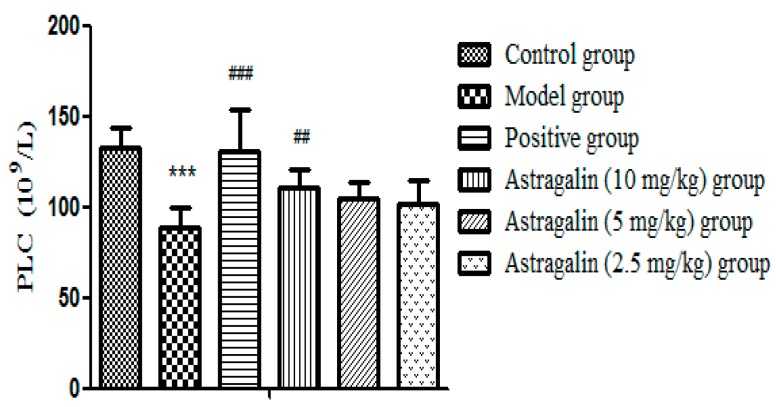
Effects of astragalin on PLC (X ± SD, *n* = 8). Compared with the control group: *** *p* < 0.001; compared with the model group: ^###^ *p* < 0.001, ^##^ *p* < 0.01.

Table 4 and Figure 4 showed that PLC in the model group was significantly lower than that in the control group (*p* < 0.001) after seven days of administration, which suggested that the model was successfully established, the number of platelets in the positive group and the astragalin (10 mg/kg) group increased significantly (*p* < 0.01). There was a slight increase in the astragalin (5 and 2.5 mg/kg) groups but no statistical difference.

#### 2.2.3. Effect of Astragalin on Plasma Coagulation Parameters In Vivo

**Figure 5:**
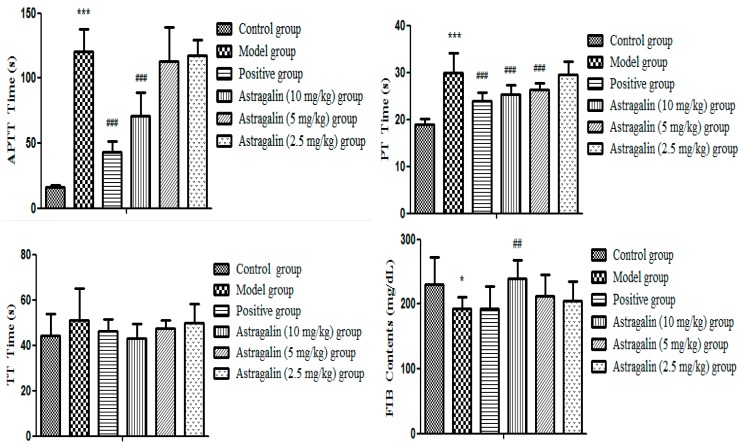
Effect of astragalin plasma coagulation parameters in vivo (X ± SD, *n* = 8). Compared with the control group: *** *p* < 0.001, * *p* < 0.05; compared with the model group: ^###^ *p* < 0.001, ^##^ *p* < 0.01.

In Table 5 and Figure 5, APTT and PT in the model group were significantly extended (*p* < 0.001) and FIB concentration was significantly decreased compared with the control group (*p* < 0.05), indicating that the model was successfully established. Compared with the model group, the positive group, the astragalin (10 and 5 mg/kg) groups all significantly shortened the effectual time of APTT and PT (*p* < 0.001), but the effect of the astragalin (10 and 5 mg/kg) groups were weaker than that of the positive group. FIB in the astragalin (10 mg/kg) group was significantly increased (*p* < 0.01), while FIB in the positive group and the astragalin (5 and 2.5 mg/kg) groups were slightly increased, but there was no statistical difference.

#### 2.2.4. Effects of Astragalin on Thromboxane B_2_ (TXB_2_) and 6-Keto Prostaglandin F_1*α*_ (6-Keto-PGF_1*α*_)

**Figure 6:**
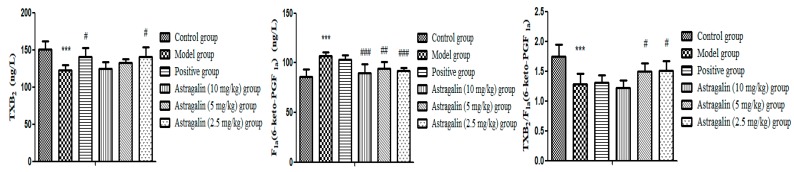
Effects of astragalin on TXB_2_ and 6-Keto-PGF_1*α*_ ($\bar{X}$ ± SD, *n* = 8)**.** Compared with the control group: *** *p* < 0.001; compared with the model group: ^###^ *p* < 0.001, ^##^ *p* < 0.01, ^#^ *p* < 0.05.

In Table 6 and Figure 6, the TXB_2_ level and TXB_2_/6-keto-PGF_1*α*_ ratio in the model group was significantly lower than that of the control group (*p* < 0.001), and 6-keto-PGF_1*α*_ was significantly higher than that of the control group (*p* < 0.001), which suggested that the model was successfully established. Compared with the model group, the contents of TXB_2_ and TXB_2_/6-keto-PGF_1*α*_ (*p* < 0.05) in astragalin (2.5 mg/kg group) were higher and it also showed a significant decrease in 6-keto-PGF_1*α*_ (*p* < 0.001). Furthermore, the effect of the astragalin (2.5 mg/kg group) on TXB_2_ was similar to that of the positive group, and the effect of the astragalin (10, 5, 2.5) mg/kg groups on 6-keto-PGF_1*α*_ was better than those of the positive group.

#### 2.2.5. Effects of Astragalin on Endothelin-1 (ET-1) and Nitric Oxide Synthase (eNOS)

**Figure 7:**
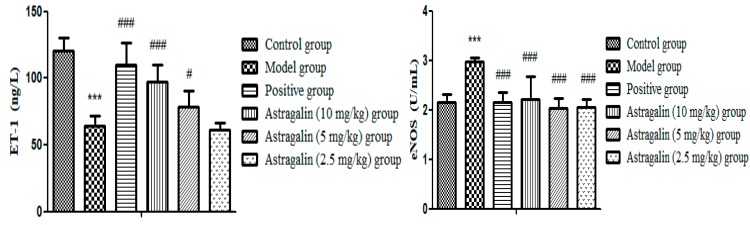
Effects of astragalin on ET-1 and eNOS (X ± SD, *n* = 8). Compared with the control group: *** *p* < 0.001; compared with the model group: ^###^ *p* < 0.001, ^#^ *p* < 0.05.

In Table 7 and Figure 7, the ET-1 of the model group was significantly lower than that of the control group (*p* < 0.001), and eNOS was significantly higher than that of the control group (*p* < 0.001), indicating that the model was successful. Compared with the model group, other groups (10 and 5 mg/kg) had significantly higher levels of ET-1 (*p* < 0.001, *p* < 0.05), and astragalin (10, 5 and 2.5 mg/kg) groups significantly lower levels of eNOS (*p* < 0.001). The effect on eNOS of the astragalin (5 and 2.5 mg/kg groups) was better than that of the positive group.

#### 2.2.6. Effects of Astragalin on Whole Blood Viscosity (WBV) and Plasma Viscosity (PV)

**Figure 8:**
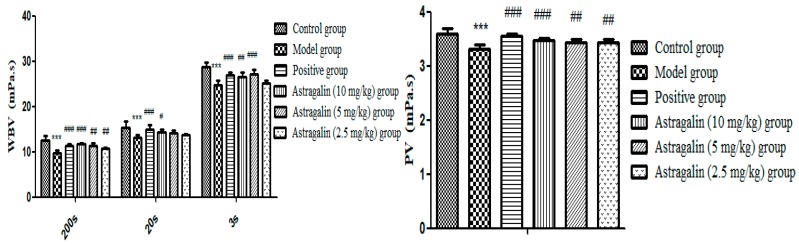
Effects of astragalin on WBV and PV (X ± SD, *n* = 8). Compared with the control group: *** *p* < 0.001; compared with the model group: ^###^ *p* < 0.001, ^##^ *p* < 0.01, ^#^ *p* < 0.05.

In Table 8 and Figure 8, the shear frequency and PV of WBV in the model group were significantly lower than that of the control group (*p* < 0.001), indicating that the modeling was successful. Compared with the model group, it was noticed that there were significant differences in the effects of the astragalin (10 and 5 mg/kg) groups on WBV with high and low shear frequency and PV (*p* < 0.001, *p* < 0.01). Compared with the model group, the astragalin (2.5 mg/kg) group could significantly increase PV (*p* < 0.01), but the effect was weaker than that of the positive group.

#### 2.2.7. Effects of Astragalin on Blood Erythrocyte Sedimentation Rate (ESR) and Packed Cell Volume (PCV)

**Figure 9:**
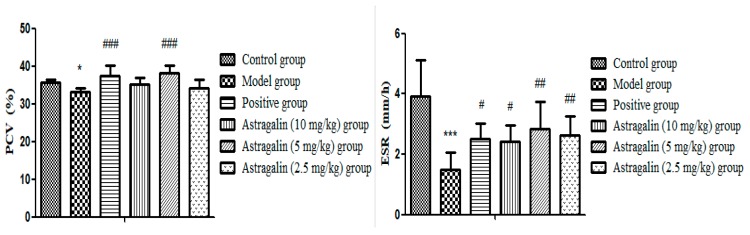
Effects of Astragalin on ESR and PCV (X ± SD, *n* = 8). Compared with the control group: *** *p* < 0.001, * *p* < 0.05; compared with the model group: ^###^ *p* < 0.001, ^##^ *p* < 0.01, ^#^ *p* < 0.05.

In Table 9 and Figure 9, PCV and ESR of the model group were significantly lower than those of the control group (*p* < 0.05, *p* < 0.001), indicating successful modeling. Compared with the model group, there was no significant difference on PCV effects in the astragalin (10 mg/kg and 2.5 mg/kg) groups, but there was significant difference on ESR (*p* < 0.05, *p* < 0.01). ESR (*p* < 0.01) and PCV (*p* < 0.001) were significantly increased in the astragalin (5 mg/kg) group compared with the model group, with the same effect as the positive group.

Normal physiological procoagulant and anticoagulant mechanisms are a complex physiological, biochemical and pathological process, including three interrelated parts: vasoconstriction and platelet response; coagulation and anticoagulation systems; and fibrinolytic system [28]. Normal coagulation of the body is mainly dependent on complete vascular wall structure and function, effective platelet quality and quantity, and normal activity of plasma coagulation factors [29]. Among them, platelets and coagulation factors play a major role [30]. Additionally, coagulation and anticoagulation systems are also affected by anticoagulation and fibrinolytic system. In order to effectively stop bleeding, there are many studies about the drugs with the activation of clotting factors and increased platelet coagulation blood [31].

However, the side effects limit their use. Natural products with procoagulant activities are current research hot spot [32,33]. In this paper, it was found that astragalin had good coagulant effect by screening of coagulation activity in vitro, which it could significantly shorten the effectual time of APTT, PT and TT at the concentrations of 5.000 mg/mL and 2.500 mg/mL (*p* < 0.001), significantly increase the content of FIB (*p* < 0.001) at concentration of 5.000 mg/mL.

And the content of astragalin in flowers of *R. chinensis* was relatively large, which had the potential value of developing as a coagulant drug. Therefore, the mechanism of astragalin on coagulant activity was further studied in vivo.

Capillary coagulation time test is widely used to screen procoagulant activity of traditional Chinese medicine, which reflects the activation process of a series of coagulation factors after blood contacting with foreign bodies in vivo, and finally plasma fibrinogen converts into fibrin resulting in blood coagulation. Moreover, how much time this process spent is dependent on the function and content of various clotting factors, which is the most intuitive parameter to judge whether a drug can promote clotting [32]. In our study, it was found that astragalin (10, 5 and 2.5 mg/kg) could significantly shorten the hemostasis time (*p* < 0.001) compared with the model group, among which the astragalin (10 mg/kg) had the same effect with the positive group. Compared with literatures, it was found that 8-*O*-acetylshanzhiside methyl ester from *L. rotata* [34], *D. draco* [35], and the flavonoid-rich fraction from the methanol extract of *O. japonicas* [36] could also shorten the coagulation time.

Platelets play an important role in blood coagulation. Their adhesion, aggregation and release play a hemostatic role, and they also provide essential membrane phospholipids for the activation of various prothrombin [37]. Astragalin (10 mg/kg) was found to promote blood coagulation by increasing the number of platelets (*p* < 0.01), which was the same as the results reported by Jia [38] on the mechanism of hemostasis of Tibetan medicine *L. rotata*.

Coagulation can generally be divided into exogenous coagulation pathway, endogenous coagulation pathway and common coagulation pathway [39]. At present, four coagulation tests (PT, APTT, TT, FIB) was used to reflect the body coagulation system in clinical [29]. PT value reflected whether the exogenous coagulation factor was abnormal APTT value reflected whether the endogenous coagulation factor was abnormal. TT value mainly reflected whether the common coagulation pathway had abnormal anticoagulation [31,40]. The content of FIB is an important indicator for the detection of cardiovascular diseases. It is mainly synthesized by the liver and can be hydrolyzed to peptides A and B under the thrombin, and finally form insoluble fibrin, so as to exert its procoagulant effect [32]. In our study, it was found that astragalin group (10 mg/kg, 5 mg/kg, 2.5 mg/kg) could significantly shorten the coagulation time (*p* < 0.001), moreover the effect of astragalin group at (10 mg/kg) was equal to the positive group. Additionally, astragalin group (10 mg/kg) could significantly reduce the effectual time of PT and APTT (*p* < 0.001), and significantly increase the content of FIB (*p* < 0.01). All of these indicated that astragalin showed procoagulant effects through endogenous coagulation pathway and exogenous coagulation pathway, which was consistent with the study on *L. rotate*, *Toddaliaasiatica* Lam., *Zea mays* L., *Sedum aizoon* L., *Gynurasegetum*(Lour.) Merr. [41,42,43,44,45].

The content of TXA_2_ could be indirectly judged by the content of TXB_2_ inside body, so as to reflect the activation degree of platelets [46]. PGI_2_ is unstable, and when released, it is rapidly transformed into the less active and more stable 6-keto-PGF_1_*_α_* [47,48]. Therefore, through measuring TXB_2_ and 6-keto-PGF_1_*_α_* in the blood, the content of TXA_2_ and PGI_2_ in the blood can be indirectly reflected, and the ratio of TXA_2_ and PGI_2_ also could reflex their hemostatic effects. In this study, the content and ratio of TXB_2_ and 6-keto-PGF_1_*_α_* in rats were measured, which proved that astragalin could promote blood coagulation by reducing the content of 6-keto-PGF_1_*_α_* and increasing the ratio of TXB_2_/6-keto-PGF_1_*_α_* in rats. In our study, it was found that astragalin (2.5 mg/kg) could increase the content of TXB_2_ and decrease the content of 6-keto-PGF_1*α*_ significantly (*p* < 0.001). This phenomenon was found in *Diospyros kaki* Thunb., which it was related to change in platelet peanut four dilute acid metabolic pathways, inhibit synthesis of platelet and release of TXA_2_, protect vascular endothelial cells, and release prostaglandin PGI, maintaining dynamic balance of TXA_2_ and PGI in the blood, inhibit vasoconstriction, and so on [49].

The synthesis of NO is controlled by its rate-limiting enzyme nitric oxide synthase (eNOS), so the content of NO can be determined by measuring the content of nitric oxide synthase (eNOS) in the blood. ET plays an important role in maintaining cardiovascular homeostasis and regulating vascular tension. The disruption of ET and NO balance will lead to many cardiovascular diseases, such as hypertension, atherosclerosis and heart failure [50]. In this study, it was proved that astragalin (5 mg/kg, 2.5 mg/kg) could promote blood coagulation by reducing the content of eNOS in rats (*p* < 0.001).

In addition, hemorheology mainly reflects changes of blood fluidity, stagnation and blood viscosity caused by changes in blood composition. It mainly includes the factors of whole blood viscosity, plasma viscosity, fibrinogen, hematocrit, and erythrocyte sedimentation rate. Abnormal hemorheology is one of the precursors of diseases such as hyperlipidemia, hypertension, coronary heart disease, and stroke [51,52]. Therefore, hemorheology is often used for diagnosis, prevention and evaluation of diseases. Erythrocyte sedimentation rate (ESR) is an important indicator in hemorheology, which refers to the sedimentation rate of erythrocyte naturally under certain conditions, usually expressed by the sedimentation rate of erythrocyte in the first hour. The volume of red blood cells in a liter of blood is called the packed cell volume (PCV). The main influence factor of whole blood viscosity is hematocrit, and it shows a positive correlation to whole blood viscosity. In our study, by measuring the whole blood viscosity and plasma viscosity in rats, it was proved that astragalin (10 mg/kg, 5 mg/kg) could increase the WBV (*p* < 0.001) and PV (*p* < 0.001) in rats, suggesting a good effect of promoting coagulation. By measuring ESR and PCV in rats, it was proved that astragalin (5 mg/kg) could play a role in promoting blood coagulation by increasing ESR and PCV in rat plasma (*p* < 0.001).

## 3. Materials and Methods

### 3.1. Materials

Breviscapine (20161103-1) was obtained from Kunming Longjin Pharmaceutical Co., Ltd. (Kunming, Yunnan, China). Yunnan Baiyao (ZJA1708) was brought from Yunnan Baiyao Group Co., Ltd. (Kunming, Yunnan, China); Protamine sulfate injection (H31020515) was gained from Shanghai First Biochemical Pharmaceutical Co., Ltd. (Shanghai, China).; PT reagent (20181225), APTT reagent (20190307), TT reagent (2019010702), and FIB assay reagent (2019010702) were manufactured by Shanghai Sun Biotechnology Co., Ltd (Shanghai, China). 6-keto-PGF_1_*_α_* ELISA kit (201907), TXB_2_ ELISA kit (201907), eNOS ELISA kit (201907), and ET-1 ELISA kit (201907) were provided by the Nanjing Senbeijia Bioengineering Institute (Nanjing, Jiangsu, China).

The flowers of *Rosa chinensis* Jacq. (20161114) were purchased from Hebei Chufeng Traditional Chinese Medicine Decoction Pieces Co., Ltd. (Anguo, Hebei, China) and were identified by professor Li Changqin (Henan University).

### 3.2. Animals

Male and female Sprague–Dawley (SD) rats (6–8 weeks, 200–250 g) and male New Zealand white rabbits (six months, 2.0–2.5 kg) were obtained from the Experimental Animal Center of Henan Province (Zhengzhou, Henan, China), and animal certificate number was SCXK 2017-0001. The animals were maintained in a 12 h light/12 h dark cycle, at 25 °C and 45–65% humidity, and fed with standard rodent diet and water adlibitum. All the animal procedures were approved by the Ethical Committee in accordance with “Instituteethical committee guidelines” for Animal Experimentation and Care. Animals were housed in standard cages. The experiment was carried out according to the guidelines of the National Institutes of Health for Care and Use of Laboratory Animals and was approved by the Bioethics Committee of Henan University.

### 3.3. Extraction and Isolation

**Figure 10:**
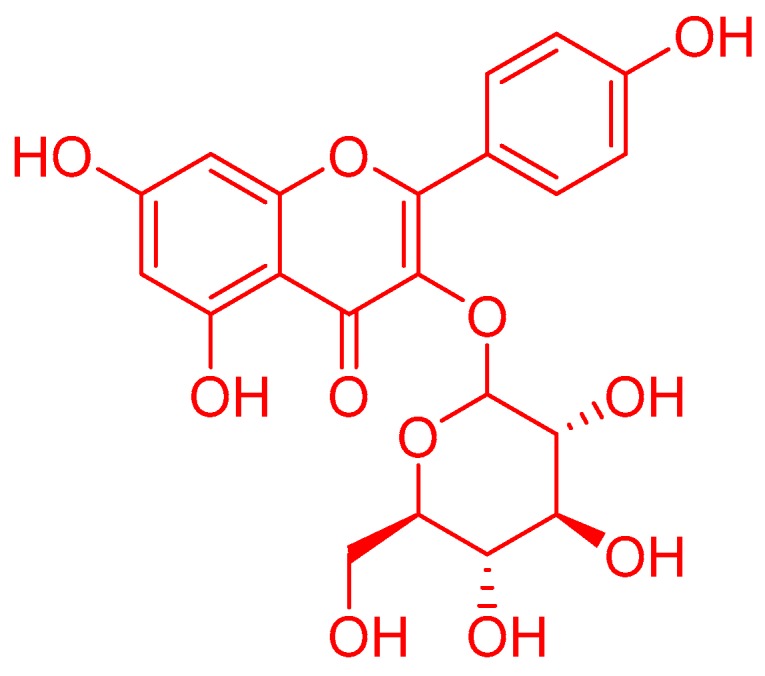
The structure of astragalin.

Dried and powered flowers of *R. chinensis* (2 kg) was extracted by petroleum ether with cold macerating at room temperature for three times, each time for one day. The residue was extracted by 70% ethanol three times for seven days, three days, and three days, respectively. The total extract was subjected to D101 macroporousresin column with gradient ethanol, and four fractions (20%, 40%, 60% and 95% ethanol) were obtained. The 40% fraction was subjected to silica gel column and eluted with CH_2_Cl_2_-MeOH (10:1–2:1) gradient to obtain raw astragalin and purified through Sephadex LH-20 column with methanol (Figure 10). 

Astragalin, yellow granular, EI-MS: *m*/*z* 448 [M]^+^, ^1^H-NMR (DMSO-d_6_, 400 MHz) δ: 12.61 (1H, s, 5-OH), 8.04 (2H, d, *J* = 8.5Hz, H-2′,6′), 6.88 (2H, d, *J* = 8.5 Hz, H-3′,5′), 6.43 (1H, d, H-8), 6.21 (1H, d, H-6), 5.46 (1H, d, *J* = 7.2Hz, H-1′′). ^13^C-NMR (DMSO-*d*_6_, 100 MHz) δ:177.5 (C-4), 164.2 (C-7), 160.0 (C-4′), 161.2 (C-5), 156.4 (C-2), 156.2 (C-9), 133.2 (C-3), 130.9 (C-2′, C-6′), 120.9 (C-1′), 115.1 (C-3′, C-5′), 104.0 (C-10), 100.8 (C-1′′), 98.7 (C-6), 93.7 (C-8), 77.5 (C-5′), 76.4 (C-3′), 74.2 (C-2′), 69.9 (C-4′), 60.8 (C-6′) [53,54].

### 3.4. Coagulation Time Test In Vitro

A blood sample (3.6 mL) was drawn from the auricular vein of the male New Zealand white rabbits and placed in a centrifuge tube containing 40 μL of 0.109 mol/L sodium citrate, mixed lightly, and centrifuged at 3000 rpm for 15min and obtained plasma. Different concentration samples of astragalin (5.000, 2.500, 1.250, 0.6250 and 0.3125 mg/mL) were tested.

#### 3.4.1. APTT Assay

Briefly, sample of astragalin (5.000, 2.500, 1.250, 0.6250, and 0.3125 mg/mL), plasma (50 μL) and APTT reagents (50 μL) were added successively and incubated at 37 °C for 5 min, and followed by adding 25 mM CaCl_2_ (50 μL). The clotting time was taken as the value of APTT. Yunnan Baiyao was used as the positive group.

#### 3.4.2. PT Assay

Sample of astragalin (5.000, 2.500, 1.250, 0.6250, and 0.3125 mg/mL) was mixed with 50 μL of plasma and incubated at 37 °C for 3 min, followed by adding 100 μL of PT reagent that was pre-incubated at 37 °C. The clotting time was recorded.

#### 3.4.3. TT Assay

Sample of astragalin (5.000, 2.500, 1.250, 0.6250, and 0.3125 mg/mL) was mixed with 100 μL of plasma and incubated for 3 min at 37 °C, then 100 μL of reagent of TT was added and the clotting time was recorded.

#### 3.4.4. FIB Assay

Plasma (100 μL) was mixed with sample of astragalin (5.000, 2.500, 1.250, 0.6250, and 0.3125 mg/mL), then buffer (350 μL) was added to obtain mixed solution. The mixed solution (200 μL) was taken and incubated at 37 °C for 3 min after blending, then 100 μL of all the enzyme solution was added and the content of FIB was recorded.

APTT, PT, TT, and FIB assays were conducted by an automatic coagulation analyzer.

### 3.5. Assays of the Procoagulant Effect of Astragalin In Vivo

Rats were randomly divided into six groups as follows: control group, model group, positive group, astragalin (10 mg/kg), astragalin (5 mg/kg), and astragalin (2.5 mg/kg) group. Each group included eight rats. Rats were caudal vein injection and administered continuously for seven days, one time per day. The control group and the model group were injected with the same blank solvent (normal saline) respectively, and the positive group was injected with protamine sulfate injection (2 mg/kg), respectively. The corresponding drugs were injected into the drug group, and the model was established on the 7th day by the following methods: on the 7th day, 5 min after the last tail vein administration, the rats in the control group were injected with normal saline, and all the other groups were injected with heparin sodium injection (500 U/kg). The rats were anesthetized with 10% chloral hydrate (300 mg/kg) after 50 min. Clotting time was measured and blood samples were taken.

#### 3.5.1. Determination of Coagulation Time 

After anesthesia, the disposable capillary was inserted into the posterior venous plexus of the eyeball on one side of the rat to take blood, and the capillary was filled with the glass capillary tube, then the glass capillary tube was placed flat, and a small section of the glass capillary tube was broken alternately from both ends every 30 s to check whether there was any coagulation. Once the coagulation occurs, break off from the other end for verification. The mean time of coagulation at both ends is the coagulation time (CT) [55].

#### 3.5.2. Number of Platelets (PLC)

After anesthesia, blood of 20 microns was collected in the inner angle of the rat′s eye with a capillary tube, diluted by 20 times with platelet dilution, and fully shaken. A total of 10 μL was absorbed and dropped into the counting pool of the blood cell counting plate, and the platelet was left to sink for 15–20 min and counted under an ordinary optical microscope [56].

#### 3.5.3. Blood Sample Collection

After anesthesia, the blood samples (5 mL) were taken from the abdominal aorta with containing sodium citrate (1:9) vacuum negative pressure pipe blood, followed by centrifuging at 3000 r/min for 15 min to obtain plasma. eNOS, ET-1, 6-keto-PGF_1*a*_ and TXB_2_ were measured. Other blood was used to measure PT, APTT, TT, and FIB. A total of 2 mL of blood was collected by a negative vacuum pressure tube containing EDTAK_2_ to determine WBV and PV, and 1.6 mL of blood was collected with a negative pressure vacuum to be containing sodium citrate (1:4) to detect ESR and PCV.

### 3.6. Statistical Analysis

All the experimental results were expressed as the mean ± standard deviation (SD). Statistical analysis was performed with the SPSS 19.0. Comparison between any two groups was evaluated using one-way analysis of variance.

## 4. Conclusions

Astragalin exerted good procoagulant effect through endogenous and exogenous coagulation pathway. It could increase the number of platelet exhibit the hemostatic activity. All these results provide us a theoretical basis for the future development and study of astragalin. Meanwhile, Astragalin is a flavonoid glycoside, riche in the flowers of *Rosa chinensis* Jacq. (1.5 g/kg). All these results indicated that astragalin can be developed as a drug. In addition, astragalin was found in the flowers of *Rosa chinensis* Jacq., which indicated us to systematic study the chemical and bioactivity of the flowers of *Rosa chinensis* Jacq., even the same genus or family to find the better compounds.

## Figures and Tables

**Table 1:** The results of coagulation four indices of samples (X ± SD, *n* = 3).

Group	APTT (s)	PT (s)	TT (s)	FIB (mg/dL)
Control group	55.33 ± 1.79	10.16 ± 0.03	31.33 ± 0.76	131.22 ± 4.18
Breviscapine group (13.30 mg/mL)	79.40 ± 0.46 ***	11.30 ± 0.25 ***	25.53 ± 0.18 ***	157.61 ± 3.18
Yunnan Baiyao group (20.00 mg/mL)	34.83 ± 0.28 ***	10.13 ± 0.06	28.73 ± 0.49 **	140.24 ± 1.06
Astragalin group (2.000 mg/mL)	41.46 ± 0.41 ***	10.30 ± 0.10	19.66 ± 0.34 ***^,&&^	160.91 ± 2.11 ***^,&&^

Compare with Control group: *** *p* < 0.001, ** *p* < 0.01; Compare with Yunnan Baiyao group: ^&&^ *p* < 0.01.

**Table 2:** Procoagulant activity of different concentrations of astragalin (X ± SD, *n* = 3).

Group	APTT (s)	PT (s)	TT (s)	FIB (mg/dL)
Control group	23.10 ± 0.10	32.43 ± 0.75	64.13 ± 0.81	83.07 ± 5.55
Yunnan Baiyao group (20.00 mg/mL)	17.30 ± 0.60 ***	30.63 ± 0.65 ***	55.46 ± 1.26 ***	91.53 ± 3.34 *
Astragalin (5.000 mg/mL)	17.50 ± 0.10 ***	28.53 ± 0.32 ***^,&&&^	52.66 ± 1.01 ***^,&&^	102.93 ± 5.83 ***^,&^
Astragalin (2.500 mg/mL)	20.77 ± 0.60 ***	29.96 ± 0.66 ***	54.20 ± 1.05 ***	98.68 ± 6.70 **
Astragalin (1.2500 mg/mL)	21.76 ± 0.57 **	30.76 ± 0.15 **	56.23 ± 0.35 ***	96.08 ± 2.71 **
Astragalin (0.6250 mg/mL)	22.60 ± 0.10	31.27 ± 0.31 *	58.40 ± 0.60 ***	94.41 ± 1.55 **
Astragalin (0.3125 mg/mL)	22.80 ± 0.26	31.83 ± 0.21	61.30 ± 1.25 **	88.71 ± 1.50

Compared with the control group: *** *p* < 0.001, ** *p* < 0.01, * *p* < 0.05; compared with the Yunnan Baiyao group: ^&&&^ *p* < 0.001, ^&&^ *p* < 0.01, ^&^ *p* < 0.05.

**Table 3:** Effects of astragalin on CT (X ± SD, *n* = 8).

Group	CT (Sec)
Control group	145.50 ± 14.01
Model group	266.25 ± 19.96 ***
Positive group	150.00 ± 21.38 ^###^
Astragalin (10 mg/kg) group	152.25 ± 18.57 ^###^
Astragalin (5 mg/kg) group	192.87 ± 15.15 ^###^
Astragalin (2.5 mg/kg) group	199.87 ± 9.99 ^###^

Compared with the control group: *** *p* < 0.001; compared with the model group: ^###^ *p* < 0.001.

**Table 4:** Effect of astragalin on PLC (X ± SD, *n* = 8).

Group	PLC (10^9^/L)
Control group	133 ± 11
Model group	89 ± 11 ***
Positive group	131 ± 23 ^###^
Astragalin (10 mg/kg) group	111 ± 10 ^##,&&^
Astragalin (5 mg/kg) group	105 ± 9 ^&&&^
Astragalin (2.5 mg/kg) group	102 ± 13 ^&&&^

Compared with the control group: *** *p* < 0.001; compared with the model group: ^###^ *p* < 0.001, ^##^ *p* < 0.01; compared with the positive group: ^&&&^ *p* < 0.001, ^&&^ *p* < 0.01.

**Table 5:** Effect of astragalin on plasma coagulation parameters in vivo (X ± SD, *n* = 8).

Group	APTT (s)	PT (s)	TT (s)	FIB (mg/dL)
Control group	16.60 ± 1.26	18.96 ± 1.21	44.10 ± 9.55	230.88 ± 41.44
Model group	120.72 ± 17.27 ***	29.94 ± 4.24 ***	51.25 ± 13.79	192.98 ± 18.16 *
Positive group	42.95 ± 8.72 ^###^	23.98 ± 1.84 ^###^	46.16 ± 5.21	193.57 ± 33.37
Astragalin (10 mg/kg) group	73.10 ± 17.68 ^###^	25.24 ± 2.02 ^###^	43.18 ± 6.09 ^&^	239.39 ± 29.19 ^##,&^
Astragalin (5 mg/kg) group	113.18 ± 26.09	26.34 ± 1.42 ^###^	47.30 ± 3.75	211.95 ± 33.36
Astragalin (2.5 mg/kg) group	117.45 ± 11.77	29.61 ± 2.75	49.75 ± 8.43	204.83 ± 30.31

Compared with the control group: *** *p* < 0.001, * *p* < 0.05; compared with the model group: ^###^ *p* < 0.001, ^##^ *p* < 0.01; compared with the positive group: ^&^ *p* < 0.05.

**Table 6:** Effect of astragalin on TXB_2_ and 6-Keto-PGF_1*α*_ (X ± SD, *n* = 8).

Group	TXB_2_(ng/L)	Fla(6-keto-PGF_1*α*_)(ng/L)	TXB_2_/Fla(6-keto-PGF_1*α*_)
Control group	150.64 ± 10.89	86.02 ± 7.29	1.74 ± 0.21
Model group	123.14 ± 6.73 ***	106.85 ± 3.95 ***	1.28 ± 0.18 ***
Positive group	140.75 ± 11.79 ^#^	103.12 ± 4.69	1.31 ± 0.12
Astragalin (10 mg/kg) group	124.93 ± 8.72	89.8 ± 9.2 ^###,^^&&&^	1.22 ± 0.12
Astragalin (5 mg/kg) group	133.04 ± 4.94	94.42 ± 6.7 ^##,^^&^	1.50 ± 0.13 ^#,^^&^
Astragalin (2.5 mg/kg) group	141.11 ± 12.65 ^#^	92.27 ± 2.76 ^###,^^&&^	1.51 ± 0.16 ^#,^^&^

Compared with the control group: *** *p* < 0.001; compared with the model group: ^###^ *p* < 0.001, ^##^ *p* < 0.01, ^#^ *p* < 0.05; Compared with the positive group: ^&&&^ *p* < 0.001, ^&&^ *p* < 0.01, ^&^ *p* < 0.05.

**Table 7:** Effects of astragalin on ET-1 and eNOS (X ± SD, *n* = 8).

Group	ET-1 (ng/L)	eNOS (U/mL)
Control group	120.49 ± 9.93	2.16 ± 0.16
Model group	63.91 ± 7.79 ***	2.97 ± 0.08 ***
Positive group	109.75 ± 16.80 ^###^	2.16 ± 0.19 ^###^
Astragalin (10 mg/kg) group	97.07 ± 13.23 ^###^	2.22 ± 0.45 ^###^
Astragalin (5 mg/kg) group	78.16 ± 12.54 ^#^	2.03 ± 0.20 ^###,&^
Astragalin (2.5 mg/kg) group	60.95 ± 5.24	2.06 ± 0.16 ^###,&^

Compared with the control group: *** *p* < 0.001; compared with the model group: ^###^ *p* < 0.001, ^#^ *p* < 0.05; compared with the positive group: ^&&^ *p* < 0.01.

**Table 8:** Effects of astragalin on WBV and PV (X ± SD, *n* = 8).

Group	WBV (mPa·s)			PV (mPa·s)
	200/s	20/s	3/s	
Control group	12.45 ± 1.03	15.32 ± 1.37	28.70 ± 0.98	3.60 ± 0.09
Model group	9.71 ± 0.62 ***	13.21 ± 0.54 ***	24.79 ± 0.91 ***	3.31 ± 0.08 ***
Positive group	11.33 ± 0.31 ^###^	14.89 ± 1.07 ^###^	26.86 ± 0.75 ^###^	3.55 ± 0.05 ^###^
Astragalin (10 mg/kg) group	11.69 ± 0.32 ^###^	14.24 ± 0.77 ^#^	26.48 ± 1.15 ^##^	3.48 ± 0.04 ^###^
Astragalin (5 mg/kg) group	11.26 ± 0.59 ^###^	14.06 ± 0.73	27.08 ± 1.06 ^###^	3.43 ± 0.07 ^##^
Astragalin (2.5 mg/kg) group	10.72 ± 0.17 ^##^	13.67 ± 0.34	25.23 ± 0.57	3.44 ± 0.05 ^##^

Compared with the control group: *** *p* < 0.001; compared with the model group: ^###^ *p* < 0.001, ^##^ *p* < 0.01, ^#^ *p* < 0.05.

**Table 9:** Effects of astragalin on ESR and PCV (X ± SD, *n* = 8).

Group	PCV (%)	ESR (mm/h)
Control group	35.95 ± 0.89	3.93 ± 1.48
Model group	33.16 ± 1.11 *	1.50 ± 0.55 ***
Positive group	37.50 ± 2.58 ^###^	2.50 ± 0.53 ^#^
Astragalin (10 mg/kg) group	35.24 ± 1.78	2.42 ± 0.53 ^#^
Astragalin (5 mg/kg) group	38.09 ± 2.02 ^###^	2.85 ± 0.90 ^##,&^
Astragalin (2.5 mg/kg) group	34.05 ± 2.33	2.64 ± 0.63 ^##^

Compared with the control group: *** *p* < 0.001, * *p* < 0.05; compared with the model group: ^###^ *p* < 0.001, ^##^ *p* < 0.01, ^#^ *p* < 0.05; compared with the positive group: ^&^ *p* < 0.05.

## Abbreviations

APTT	activated partial thromboplastin time
PT	prothrombin time
TT	thrombin time
FIB	plasma fibrinogen
6-keto-PGF_1*α*_	6-keto prostaglandin F_1*α*_
eNOS	nitric oxide synthase
TXB_2_	thromboxane B_2_
ET-1	endothelin-1
ESR	erythrocyte sedimentation rate
PCV	packed cell volume
WBV	whole blood viscosity
PV	plasma viscosity
TXA_2_	thromboxane A_2_
PGI_2_	prostaglandin I_2_
NO	nitric oxide
SD	Sprague–Dawley
CT	coagulation time
PLC	platelet count
EI-MS	electronimpact mass spectrometry
HPLC	high performance liquid chromatography
